# Visualization of the lenticulostriate arteries, long insular arteries, and long medullary arteries on intra-arterial computed tomography angiography with ultrahigh resolution in patients with glioma

**DOI:** 10.1007/s00701-023-05794-1

**Published:** 2023-09-20

**Authors:** Yoshinari Osada, Masayuki Kanamori, Shin-Ichiro Osawa, Shingo Kayano, Hiroki Uchida, Yoshiteru Shimoda, Shunji Mugikura, Teiji Tominaga, Hidenori Endo

**Affiliations:** 1 Department of Neurosurgery, Tohoku University Graduate School of Medicine, 1-1 Seiryo-Machi, Aoba-Ku, Sendai, Miyagi 980-8575 Japan; 2 Department of Radiological Technology, Tohoku University Hospital, Tohoku University, Sendai, Miyagi Japan; 3 Department of Diagnostic Radiology, Tohoku University Graduate School of Medicine, Sendai, Japan; 4 Department of Image Statistics, Tohoku Medical Megabank Organization, Tohoku University, Sendai, Japan

**Keywords:** Ultrahigh-resolution computed tomography, CT angiography, Long insular arteries, Long medullary arteries, Pyramidal tract, Insulo-opercular glioma

## Abstract

**Purpose:**

The anatomical association between the lesion and the perforating arteries supplying the pyramidal tract in insulo-opercular glioma resection should be evaluated. This study reported a novel method combining the intra-arterial administration of contrast medium and ultrahigh-resolution computed tomography angiography (UHR-IA-CTA) for visualizing the lenticulostriate arteries (LSAs), long insular arteries (LIAs), and long medullary arteries (LMAs) that supply the pyramidal tract in two patients with insulo-opercular glioma.

**Methods:**

This method was performed by introducing a catheter to the cervical segment of the internal carotid artery. The infusion rate was set at 3 mL/s for 3 s, and the delay time from injection to scanning was determined based on the time-to-peak on angiography. On 2- and 20-mm-thick UHR-IA-CTA slab images and fusion with magnetic resonance images, the anatomical associations between the perforating arteries and the tumor and pyramidal tract were evaluated.

**Results:**

This novel method clearly showed the relationship between the perforators that supply the pyramidal tract and tumor. It showed that LIAs and LMAs were far from the lesion but that the proximal LSAs were involved in both cases. Based on these results, subtotal resection was achieved without complications caused by injury of perforators.

**Conclusion:**

UHR-IA-CTA can be used to visualize the LSAs, LIAs, and LMAs clearly and provide useful preoperative information for insulo-opercular glioma resection.

**Supplementary information:**

The online version contains supplementary material available at 10.1007/s00701-023-05794-1.

## Introduction

Insular and opercular glioma resection is challenging to perform due to the surrounding eloquent cortex and white matter network that are responsible for language and motor function [14, 25, 26]. Furthermore, caution should be observed as arteries supplying the pyramidal tract, including the lenticulostriate arteries (LSAs), long insular arteries (LIAs), and long medullary arteries (LMAs), can be perforated [6, 8, 10, 12, 14, 17, 23, 24, 26]. The LSAs supply the putamen, globus pallidus and internal capsule, and adjacent corona radiata [4, 17, 28]. Injury during insular glioma resection results in permanent motor deficits [8, 10, 12, 14, 17, 24, 26]. To prevent this complication, attempts have been made to visualize the LSAs on three-dimensional (3D) contrast-enhanced time-of-flight magnetic resonance (MR) angiography with a 3 T or 7 T scanner [22, 23], T2-weighted imaging (T2WI) with a 7 T scanner [2], flow-sensitive black-blood MR angiography [20], and cone-beam CT scan [16]. The LIAs originate from the M2, M2–M3 junction, or M3 of the middle cerebral artery (MCA). Moreover, the LIAs supplying the pyramidal tract commonly penetrate the insular cortex on the top of the insular long gyri or in the superior limiting sulcus and run to the pyramidal tract [3, 7]. The LMAs supplying the pyramidal tract arise from the proximal M4 segment of the MCA and run linearly to the lateral ventricle [1]. To prevent injury in these perforators, previous studies have reported the temporary occlusion of the LIAs under motor-evoked potential monitoring [17] and the high-risk locations related to infarctions in the territory of the LIAs and LMAs via image analysis of insulo-opercular glioma [12, 26]. However, the current imaging technique cannot visualize either LIAs or LMAs directly because of their thin diameter (500 and 100–200 µm, respectively) [1, 3].

To visualize LIAs and LMAs, a novel method combining the intra-arterial injection of contrast medium and ultrahigh-resolution CT angiography (UHR-IA-CTA) was developed. Recently, a UHR-CTA scanner with a detector row width of 0.25 mm and a matrix of 1024 × 1024 has been available in clinical practice [9]. This scanner can improve spatial resolution from 400–450 to nearly 150–200 µm [13], and UHR-IA-CTA has been applied for the identification of the LSA [18] and the artery of Adamkiewicz [29] and the postoperative evaluation for neck clipping of aneurysm [11]. However, the LIAs and LMAs were not identified on UHR-IV-CTA due to the simultaneous depiction of the intraparenchymal veins and low contrast resolution for identifying minute vessels [23]. To improve these issues, a previous study reported the usefulness of the intra-arterial, but not intravenous, injection of contrast medium in identifying the artery of Adamkiewicz [19]. Based on these data, UHR-IA-CTA can be a promising tool as it can improve the visualization of perforating arteries.

Herein, we report our initial experience with the visualization of the LSAs, LIAs, and LMAs with UHR-IA-CTA in patients with insulo-opercular glioma.

## Methods

### Patients

The current study was approved by the Institutional Ethical Review Board of our hospital. A written informed consent was obtained from all patients.

#### UHR-IA-CTA

The procedure was divided into four, which were as follows: the intra-arterial injection of contrast medium with the endovascular procedure, the acquisition using UHR-IA-CTA images, the acquisition of MR images, and image processing.

Endovascular procedure: In a suite for angiogram, a 4-French sheath was introduced to the right radial arteries. Under systemic heparinization to double PT-INR from baseline, a 4-French catheter (CX 115 cm; Gadelius Medical, Tokyo) was introduced over a 0.035-in. Radifocus guidewire (Thermo, Tokyo) to the cervical segment of the internal carotid arteries proximal to the targeted area. Conventional digital subtraction angiography was performed with an infusion rate of 3 mL/s, and 5 mL of contrast medium was administered with an autoinjector. The capillary filling and time-to-peak contrast from infusion were evaluated. After sealing the puncture site, the patient was transferred to the UHR-CT unit without moving the catheter tip.

UHR-IA-CTA: The catheter was connected to the autoinjector, and air was cautiously ejected. UHR-IA-CTA was performed using a 160-detector row UHR-CT scanner system with a 1024 × 1024 matrix and a slice thickness of 0.25 mm (Aquilion Precision; Canon Medical Systems, Otawara, Japan). Nonionic contrast medium with an iodine concentration of 300 mgI/mL iohexol (Omnipaque 350; GE Healthcare Pharma, Tokyo, Japan) and iopamidol (Iopamiron 370; Bayer Healthcare, Osaka, Japan) was administered at an infusion rate of 3 mL/s. The total volume administered with an autoinjector was 9 mL for 3 s. Based on the time-to-peak, the delay from injection to scanning was set to 1.5–2.0 s. The following scanning parameters were used in UHR-IA-CTA: tube voltage, 120 kV; tube current, 240 mA; collimation, 0.25 mm × 160; beam pitch factor, 0.569; rotation speed, 0.75 s; slice thickness, 0.25 mm; slice interval, 0.25 mm; scanning field of view (FOV), 320 mm; and reconstruction kernel of forward-projected model-based iterative reconstruction solution algorithm. The image matrix size was 1024 × 1024, and the display FOV was 200 mm. To evaluate the perforating arteries from the origin to the pyramidal tract, the scan coverage was set for each individual involving the supra-clinoid segment of the internal carotid arteries and the M1, M2, M3, and proximal M4 segment of the MCAs.

Acquisition of MR images: To estimate the distribution of glioma and the location of the pyramidal tract, 3D T1-weighted images (T1WI) before and after the administration of gadobutrol (Gd-T1WI) (Gadovist; Bayer Healthcare, Osaka, Japan) in Cases 1 and 2 or T2WI in Case 2 and diffusion tensor images (DTI) were obtained. Briefly, MR images were collected using a 3.0 Tesla unit (Intera Achieva 3.0 T Quasar Dual; Philips Medical Systems, Best, the Netherlands). For 3D-T1WI and 3D-Gd-T1WI, Magnetization-Prepared Rapid Gradient Echo was used with the following parameters: TR/TE, 6.7/3.1 ms; slice thickness, 0.9 mm; FOV, 240 × 223 mm; matrix size, 268 × 248; and 450 sagittal slices. For 3D-T2WI, Volume ISotropic T2w Acquisition was applied with the following parameters: TR/TE, 3000/180 ms; slice thickness, 1.3 mm; FOV, 224 × 202 mm; matrix size, 400 × 360; and 300 transverse slices. For DTI, we used single-shot spin-echo echo-planar sequences with the following parameters: b, 800 s/mm^2^; 15 directions; TR/TE, 5601/63 ms; slice thickness, 3 mm; FOV, 224 × 224 mm; matrix size, 128 × 126; and 50 transverse slices.

**Fig. 1:**
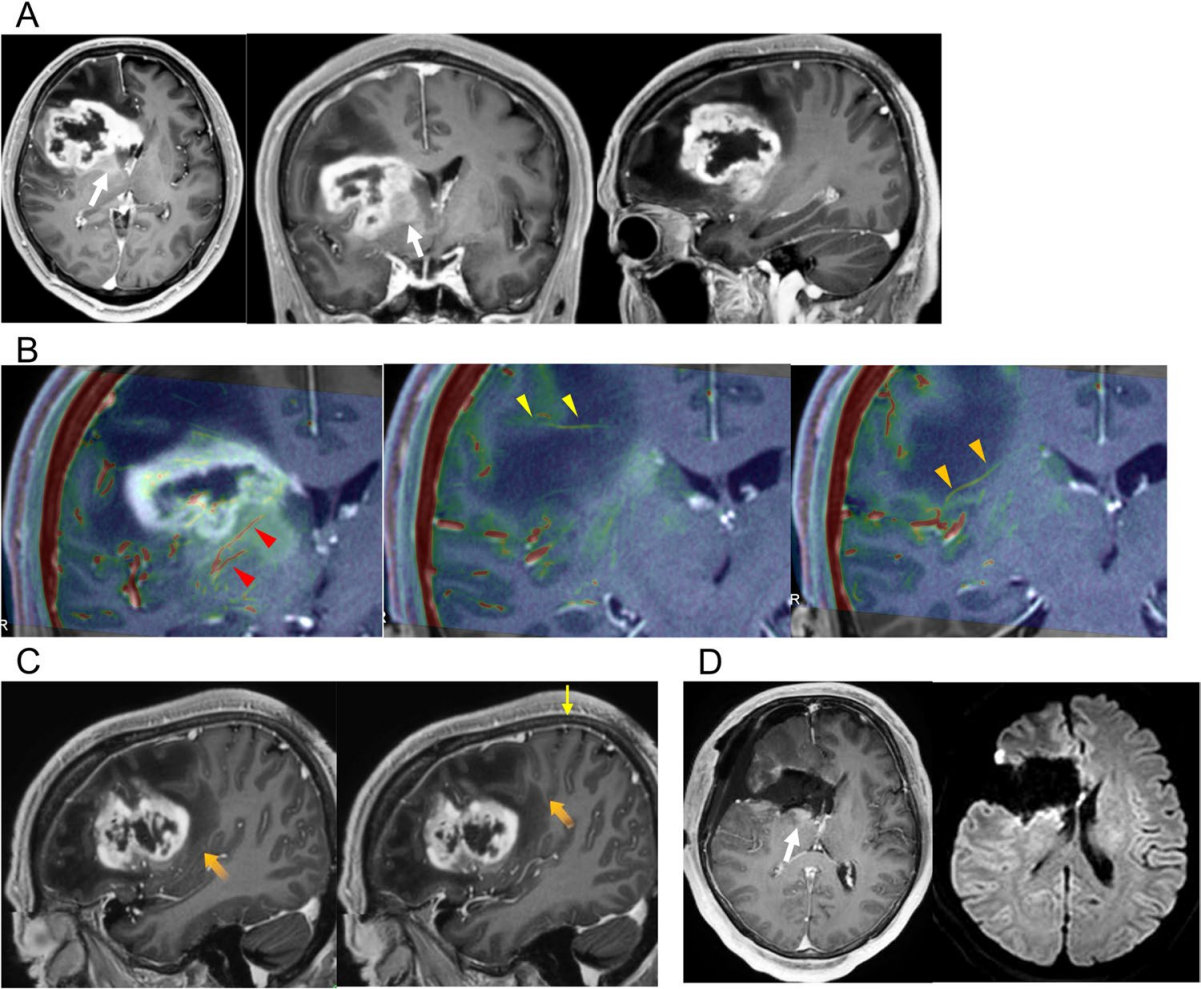
Preoperative assessment of the anatomical association between the long insular arteries (LIAs), long medullary arteries (LMAs), and lenticulostriate arteries (LSAs) and tumor and postoperative magnetic resonance (MR) imaging in Case 1. **A** Preoperative axial (left panel), coronal (middle panel), and sagittal (right panel) T1-weighted MR imaging after the administration of gadolinium (Gd-T1WI) showing the strongly enhanced lesion at the right frontal operculum and insula and the weakly enhanced lesion at the right basal ganglia (arrows). **B** The coronal slice with the LSAs (left panel), LMAs (middle panel), and LIAs (right panel) showing that LSAs (red arrowheads) were close to the strongly enhanced lesion and encased in the weakly enhanced lesion and that the LMA (yellow arrow) and LIA (orange arrowheads) were far from the enhanced lesion. **C** Sagittal Gd-T1WI showing the origin of the LIA (orange arrow in the left panel) and LMA (orange arrow in the right panel), as shown in Fig. 1C. The yellow arrow indicates the central sulcus. The LIA and LMA originated from the posterior region of the insula and the bottom of the central sulcus, respectively. **D** Postoperative axial Gd-T1WI (left panel) and diffusion-weighted MR imaging (right panel) showing the subtotal resection of the enhanced lesion without infarction due to injury in the perforating arteries

**Fig. 2:**
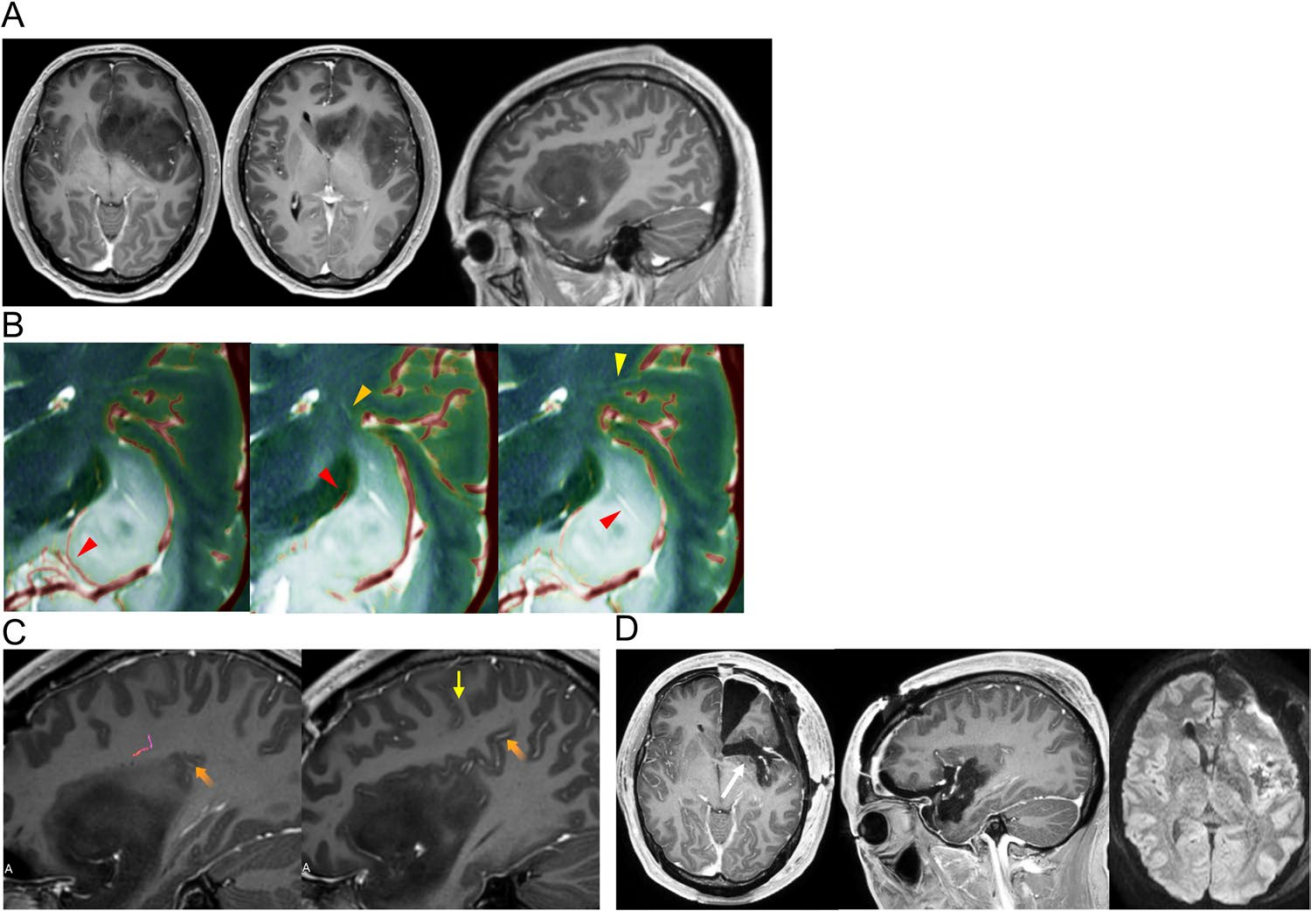
Preoperative assessment of the anatomical association between the long insular arteries (LIAs), long medullary arteries (LMAs), and lenticulostriate arteries (LSAs) and the tumor and postoperative magnetic resonance (MR) imaging in Case 2. **A** Preoperative axial (left and middle panel) and sagittal (right panel) T1-weighted MR imaging after the administration of gadolinium (Gd-T1WI) showing the lesion at the left subcallosal area and insula with the small enhanced area. **B** The coronal slice with LSAs (all panels), LIAs (middle panel), and LMAs (right panel) showing that the distal LSA (red arrows in the middle and right panel), LIA (orange arrowhead), and LMA (yellow arrow) were located outside from the hyperintense lesion. However, proximal LSA (red arrowhead in the left panel) was encased in the lesion. **C** Sagittal Gd-T1WI showing the origin of the LIA (orange arrow in the left panel) and LMA (orange arrow in the right panel), as shown in Fig. 2C. The yellow arrow indicates the central sulcus. The LIA and LMA originated from the posterior region of the insula and the sulcus within the supramarginal sulcus, respectively. **D** Postoperative axial (left panel) and sagittal (middle panel) Gd-T1WI and diffusion-weighted MR imaging (right panel) showing the subtotal resection of the lesion without infarction due to perforating injury. The arrow indicates the residual region at the anterior perforating substance where the LSA was encased by the tumor

Image processing: Image processing was performed using a commercially available workstation (Ziostation2; Ziosoft, Tokyo, Japan). Using the coronal slices parallel to the central arteries on UHR-IA-CTA, the LIAs, LMAs, and LSAs were visualized (Supplemantal Fig. 1A and 2A). Then, the set of T2WI, T1WI, Gd-T1WI, DTI, and UHR-IA-CTA images were fused. On the workstation, tractography of the pyramidal tract was performed. The single voxel of interest was set in the ventral craniocaudal tract in the ipsilateral midbrain on a 3D anisotropy contrast-enhanced image. The minimum streamline length and FA cutoff were set to 80 mm and 0.10, respectively. The whole distribution of the vessels (Supplemantal Fig. 1B and 2B) and the spatial association between the vessels and the pyramidal tract, MCA, and tumors (Supplemantal Fig. 1C and 2C, Supplemental movie files 1 and 2) were evaluated using 20- and 2-mm-thick slab fusion images, respectively. To assess the anatomical association between the perforating arteries and tumor, the images of the color map of UHR-IA-CTA and Gd-T1WI or T2WI were fused.

## Results

### Illustrative cases

#### Case 1

A 66-year-old male patient presented with lethargy and left hemiparesis for 1 month. Upon admission, he had left hemiparesis. MR images revealed a strongly enhanced lesion at the right frontal operculum and insula and a weakly enhanced lesion at the basal ganglia (Fig. 1A). To estimate the risk of perforator injury, the course of perforating arteries was examined via UHR-IA-CTA. On 20-mm-thick UHR-IA-CTA, the candidate vessels for LSAs, LIAs, and LMAs were identified (Supplemantal Fig. 1B). On sequential 2-mm slab fusion images between UHR-IA-CTA and tractography of the pyramidal tract (Supplemantal Fig. 1C and Supplemental movie file 1), the perforating vessels originated from the M2–M3 junction and M4 and run toward the pyramidal tract. The vessels marked by orange and yellow arrowheads were considered as LIAs and LMAs, respectively (Supplemantal Fig. 1C and Supplemental movie file 1). Meanwhile, the vessels not connecting directly to MCA was considered as submedullary vein draining into the peri-insular sulcus vein around the superior limiting sulcus (Supplemantal Fig. 1C and Supplemental movie file 1). On the fusion image between UHR-IA-CTA and Gd-T1WI (Fig. 1B), the anatomical association between the candidate vessels supplying the pyramidal tract and tumor could be examined. The LIAs and LMAs supplying the pyramidal tract were far from the enhanced lesion. Meanwhile, the LSAs were encased in the weakly enhanced lesion at the basal ganglia (Fig. 1B). The LIA originated from the posterior region of the insula (Fig. 1C). Based on these findings, the enhanced lesion except around the LSA could be safely resected. Subtotal tumor resection was performed while preserving the perforators (Fig. 1D). The histological diagnosis was isocitrate dehydrogenase (IDH) wild-type glioblastoma [15]. The patients received 60 Gy of radiation therapy to the local site and temozolomide.

#### Case 2

A 38-year-old male patient presented with acute and transient episodes of hypesthesia of the right upper and lower limbs for 3 months. Upon admission, he was alert and had no focal neurological deficits but had attacks of hypesthesia several times a day. MR images revealed a hypointense lesion with a slightly enhanced area at the left insula and subcallosal area on Gd-T1WI (Fig. 2A). The insular lesion was located at zones I and II based on the classification of Sanai and Berger [25], and it extended to the posterior region of the superior limiting sulcus (Fig. 2A). Because of its large volume, the patient underwent two-stage surgery. After resecting the left frontal and subcallosal lesion, which was histologically diagnosed as IDH-mutant, grade 3 astrocytoma [15], we planned to resect the insular lesion. To estimate the risk of perforator injury, the courses of perforating arteries were examined on UHR-IA-CTA. On UHR-IA-CTA with 20-mm-thick slabs, the candidate vessels for LIAs and LMAs could be identified. Nevertheless, the findings were not definitive (Supplemantal Fig. 2B). These vessels originated from the M2–M3 junction and M4 on the 2-mm-thick fusion slab images of UHR-IA-CTA and fiber tracking of the pyramidal tract; the vessels marked by orange and yellow arrowheads were considered as LIA and LMA, which supply the pyramidal tract (Supplemantal Fig. 2C and Supplemental movie file 2). On fusion images between UHR-IA-CTA with 2-mm-thick slabs and T2WI, the distant LSAs, LIA, and LMA were located outside the tumor. Meanwhile, the proximal LSAs were encased in the tumor (Fig. 2B). The LIA originated from the posterior region of the insula and the LMA from the anterior parietal artery in the sulcus within the supramarginal sulcus (Fig. 2C). Based on these findings, the insular lesion except for the area around the proximal LSAs could be resected. Subtotal tumor resection was performed while preserving these perforators (Fig. 2D). The residual lesion did not grow after 60 Gy of radiation therapy to the local site and nimustine hydrochloride [27].

## Discussion

This is the first report showing that IA-CTA on UHR-CT fused with Gd-T1WI or T2WI and tractography can accurately assess the association between the LSAs, LIAs, and LMAs supplying the pyramidal tract and tumor.

The novel method in this study provided a spatially high resolution and signal-to-noise ratio. For example, the branching, change in size, tortuosity, and the density of the LSA branches around the internal capsule and the LIA and LMA branches [21] were clearly observed in Case 1. Thus far, no imaging modalities in clinical practice have shown LSA branching, and this is the first report revealing LIA and LMA branching.

To improve LIA and LMA visualization, we devised a method to perform IA-CTA and analyze the images. At IA-CTA, it is important to optimize the timing between injection from the catheter placed in the carotid artery and imaging for visualizing the perforating artery only. Angiography was performed before CTA to optimize the timing of the early arterial phase after injection. In analyzing the images, the method for processing the images was also optimized. The 3D images from UHR-IA-CTA were analyzed in coronal slices along the LIA and LMA. In previous reports, the LIAs and LMAs run perpendicular to the M2–M3 and M4 toward the ventricles, respectively [12, 20]. Therefore, using a coronal slice parallel to the central artery, the LIA and LMA along the long axis could be evaluated, as shown in Supplemantal Figs. 1C and 2C.

In the process of visualizing the perforating arteries, the discrimination between the LIA or LMA and the veins was the most challenging issue. The course of the submedullary veins draining to the insular or peri-insular veins was similar to that of the LIA and LMA. In this study, the vessels branching directly from the M2, M3, and M4, and those with a clear enhancement, as shown in two cases, on 2-mm-thick slab images of UHR-IA-CTA were considered as the perforating artery. Meanwhile, the vaguely enhanced vessels connected to the peri-insular or insular veins along the superior limiting sulcus or on the sulcus of the insula were considered as the submedullary veins, as reported in a previous study [5] (Supplemantal Fig. 1C and Supplemental Fig. 3A). The characteristics of the vessels identified as the LIA and LMA in this study were consistent with those in previous reports regarding the presence of the perivascular space and their origins. The perivascular space around the LIA and LMA were found in Case 2 (Supplemental Fig. 3B). This was the specific finding of the perforating arteries. However, it was not observed at the medullary veins [2, 26]. The LIA originated from the posterior of the insula in both cases, as reported in previous studies [8, 12, 14]. Although postcentral gyrus resection is a risk factor due to damage in the radiologically invisible LMA [26], the origin of LMA has not been identified yet. This is the first report showing the origin of LMA using a clinical radiographical approach.

Recently, some medical device manufacturers have developed high-resolution 3D angiography featuring a slightly better resolution than that of UHR-CT. In the present study, the XYZ resolution obtained using UHR-CT was 0.15 mm, whereas that obtained using high-resolution 3D angiography in Azurion 7 B20/15 (Phillips, Amsterdam, Netherlands) and ARTIS icono D-Spin (Siemens, München, Germany) was 0.1 mm. Although the images produced by high-resolution 3D angiography can be fused with MR images, including Gd-T1WI, T2WI, and tractography on a limited workstation, such as Brainlab Elements (Brainlab, München, Germany), the fusion images between that from high-resolution 3D angiography and MR images cannot be created with other software. Considering the fact that it does not require other medical devices or the transfer of patients, high-resolution 3D angiography has an advantage if a workstation is developed in the future. Furthermore, the findings of this study are of significance with respect to the proposal of a new technique for the depiction of LIA and LMA.

Despite efforts in the acquisition of imaging and image analysis, there are still issues that should be overcome. In the acquisition of imaging, the optimization of the time between the injection of contrast medium via the catheter and the start of imaging has been addressed on a case-by-case basis. Since the time-to-peak is longer in IV-CTA than in IA-CTA, the timing of imaging can be automatically determined by detecting the arrival of the contrast medium. However, using this method, it is challenging to start imaging automatically because of the narrow time window caused by the proximity between the injection site and the imaging site. In the imaging analysis, two issues were considered. First, it is challenging to distinguish normal from abnormal vessels in the case of arteriovenous shunts and vascular proliferation, which has been frequently observed in glioblastoma. The identification of LIAs and LMAs close to the tumor is significantly clinically important. Second, we could not create the simultaneous fusion image showing the relationship between the perforator, pyramidal tract, and tumor. Thus, we first need to identify the LSA, LIA, and LMA that supply the pyramidal tract on the fusion image between UHR-IA-CTA and tractography and determined the relationship between the identified perforators and tumor on the fusion image between UHR-IA-CTA and Gd-T1WI or T2WI. Moreover, a limitation in the validation of the results based on the intraoperative findings was observed. Based on the findings from UHR-IA-CTA, we concluded that LIA and LMA were not involved in the tumor and that the tumor resection close to these perforators was safe and feasible. Consistent with this judgment, the lesions were resected safely without exposing the LIAs and LMAs. In contrast, since the lesion that involved LSA in Case 1 and proximal LSA in Case 2 was considered to be unresectable, the involved LSA was not confirmed in either of the cases. Although distal LSAs, which run medial to the tumor in Case 2, could have been confirmed intraoperatively, this vessel was not exposed intraoperatively due to the potential damage that can be caused by LSAs. With the increasing number of cases, the issues not addressed in this study will become clearer, and more cases should be gathered.

## Conclusion

UHR-IA-CTA can facilitate a detailed evaluation of the LSAs, LIAs, and LMAs and can provide useful information to prevent injury in the perforating arteries during insulo-opercular glioma resection.

### Supplementary information

Below is the link to the electronic supplementary material.

Supplementary file1 The sequential fusion images between intra-arterial computed tomography angiography using ultrahigh-resolution computed tomography with 2-mm-thick slabs and tractography of the pyramidal tract in Case 1. The orange, yellow, and red arrowheads indicate the long insular arteries, long medullary arteries, and lenticulostriate arteries supplying the pyramidal tract, respectively. Perforating arteries originated from the middle cerebral artery or it branches directly. Meanwhile, the vessels connecting to the peri-insular sulcus veins was considered as the veins (blue arrowhead).(MP4 8490 KB)

Supplementary file2 The sequential fusion images between UHR-IA-CTA with a 2-mm-thick slabs and tractography of the pyramidal tract in Case 2. The orange, yellow, and red arrowheads indicate the long insular artery, long medullary artery, and lenticulostriate arteries supplying the pyramidal tract.(MP4 7236 KB)

Supplementary file3 Intra-arterial computed tomography (CT) angiography using ultrahigh-resolution CT (UHR-IA-CTA) in Case 1. A. Sagittal T1-weighted magnetic resonance (MR) imaging after the administration of gadolinium (Gd-T1WI) (left panel) and 20-mm-thick slab imaging of UHR-IA-CTA (right panel), thereby showing the coronal slice (yellow dashed line) for identifying the long insular artery (LIA) and long medullary artery (LMA). Based on the central sulcus (yellow arrows) on sagittal Gd-T1WI, the central artery (white arrowhead) was identified, and the coronal slice parallel to the central arteries was made on UHR-IA-CTA. B. The coronal slice with the lenticulostriate arteries (LSAs) (left panel) and with the LIA and LMA (right panel) of UHR-IA-CTA with 20-mm-thick slabs showing the distribution of the LSAs (red arrowheads), LIAs (orange arrowhead), and LMAs (yellow arrowhead). There were tortuous and dilated tumor vessels in the frontal lobe and insula, and LSAs were displaced medially by the tumor. The asterisk indicates LSA branching in the putamen and caudate nucleus. The white arrows in the right panel indicates the internal capsule. The branching, change in size, tortuosity, and density of LSA branches around the internal capsule were noted. C. The coronal slice with the LIAs and LMAs of UHR-IA-CTA with 2-mm-thick slabs, which fused to tractography of the pyramidal tract showing the association between the LIAs (orange arrowheads) and LMAs (yellow arrowheads) supplying the pyramidal tract (purple, blue and green), M2, M3, and M4 (red arrows). LIAs and LMAs directly originated from the M2–M3 junction and M4 and supplied the pyramidal tract. The vessels not connecting the middle cerebral arteries with vague enhancement were considered as the subependymal veins (blue arrowheads) that drain into the peri-insular sulcus or insular vein at the superior limiting sulcus.(TIF 2456 KB)

Supplementary file4 Intra-arterial computed tomography (CT) angiography using ultrahigh-resolution CT (UHR-IA-CTA) in Case 2. A. Sagittal T1-weighted MR imaging after the administration of gadolinium (Gd-T1WI) (left panel) and 20-mm-thick slab imaging of UHR-IA-CTA (right panel) showing the coronal slice (dashed line) for identifying the long insular arteries (LIA) and long medullary arteries (LMA). Based on the central sulcus (yellow arrows) on sagittal Gd-T1WI, the central artery (white arrowhead) was identified, and the coronal slice parallel to the central arteries was made on UHR-IA-CTA. B. The coronal slice with the lenticulostriate arteries (LSAs) (red arrowhead), long medullary artery (LMAs) (yellow arrowheads in the left panel), and long insular artery (LIA) (orange arrowheads in the right panel) of UHR-IA-CTA with 20-mm-thick slabs showing the distribution of the LSA, LMA, and LIA. C. The coronal slice with the LMA (yellow arrowheads in the left panel), LIA (orange arrowheads in the middle panel), and proximal LSAs (red arrowhead in the middle and right panel) of UHR-IA-CTA with 2-mm-thick slab, which fused to tractography of the pyramidal tract, showing the association between the perforating arteries and pyramidal tract (purple, blue and green) and the middle cerebral arteries (red arrows). The LSAs, LIAs, and LMAs supplying the pyramidal tract originated from the M1, M2,, and M3 junction, and M4, respectively.(TIF 2390 KB)

Supplementary file5 The discrimination of the perforating arteries from the veins. A. Sagittal imaging (left panel) and the coronal slice parallel to the central artery (right panel) of intra-arterial computed tomography (CT) angiography using ultrahigh-resolution CT (UHR-IA-CTA) with 20-mm-thick slabs (left panel) showing the peri-insular sulcus veins along the superior limiting sulcus. These vaguely enhanced vessels were connected to the peri-insular sulcus veins along the superior limiting sulcus, but not to the M2, M3, or M4 (blue arrowheads). B. UHR-IA-CTA with 2-mm-thick slabs (upper panels) and T2-weighted imaging (T2WI) (lower panels) that is the identical cross image to UHR-IA-CTA in Case 2 showing the perivascular space around the lenticulostriate arteries (red arrowheads), long medullary arteries (yellow arrowhead), and long insular arteries (orange arrowhead) on T2WI.(TIF 2494 KB)

## Data Availability

Not applicable.

## References

[1] Akashi T, Takahashi S, Mugikura S (2017). Ischemic white matter lesions associated with medullary arteries: classification of MRI findings based on the anatomic arterial distributions. AJR Am J Roentgenol.

[2] Bouvy WH, Biessels GJ, Kuijf HJ, Kappelle LJ, Luijten PR, Zwanenburg JJ (2014). Visualization of perivascular spaces and perforating arteries with 7 T magnetic resonance imaging. Invest Radiol.

[3] Delion M, Mercier P (2014). Microanatomical study of the insular perforating arteries. Acta Neurochir (Wien)..

[4] Duffau H (2009). A personal consecutive series of surgically treated 51 cases of insular WHO Grade II glioma: advances and limitations. J Neurosurg.

[5] Gogia B, Chavali LS, Lang FF (2018). MRI venous architecture of insula. J Neurol Sci.

[6] Gogos AJ, Young JS, Morshed RA (2020). Triple motor mapping: transcranial, bipolar, and monopolar mapping for supratentorial glioma resection adjacent to motor pathways. J Neurosurg.

[7] Ikegaya N, Takahashi A, Kaido T (2018). Surgical strategy to avoid ischemic complications of the pyramidal tract in resective epilepsy surgery of the insula: technical case report. J Neurosurg.

[8] Iwasaki M, Kumabe T, Saito R (2014). Preservation of the long insular artery to prevent postoperative motor deficits after resection of insulo-opercular glioma: technical case reports. Neurol Med Chir (Tokyo).

[9] Kakinuma R, Moriyama N, Muramatsu Y (2015). Ultra-high-resolution computed tomography of the lung: image quality of a prototype scanner. PLoS One.

[10] Kawaguchi T, Kumabe T, Saito R (2014). Practical surgical indicators to identify candidates for radical resection of insulo-opercular gliomas. J Neurosurg.

[11] Kayano S, Ito A, Endo T (2022). Efficacy of ultra-high-resolution computed tomographic angiography for postoperative evaluation of intracranial aneurysm after clipping surgery: a case report. Surg Neurol Int.

[12] Kumabe T, Higano S, Takahashi S, Tominaga T (2007). Ischemic complications associated with resection of opercular glioma. J Neurosurg.

[13] Kwan AC, Pourmorteza A, Stutman D, Bluemke DA, Lima JAC (2021). Next-generation hardware advances in CT: cardiac applications. Radiology.

[14] Lang FF, Olansen NE, DeMonte F (2001). Surgical resection of intrinsic insular tumors: complication avoidance. J Neurosurg.

[15] Louis DN, Perry A, Wesseling P (2021). The 2021 WHO classification of tumors of the central nervous system: a summary. Neuro Oncol.

[16] Matsushige T, Hashimoto Y, Ogawa T (2022). The impact of high-resolution cone-beam CT findings on decision-making for the treatment of unruptured middle cerebral artery aneurysms. Neurosurg Rev.

[17] Moshel YA, Marcus JD, Parker EC, Kelly PJ (2008). Resection of insular gliomas: the importance of lenticulostriate artery position. J Neurosurg.

[18] Murayama K, Suzuki S, Nagata H (2020). Visualization of lenticulostriate arteries on CT angiography using ultra-high-resolution CT compared with conventional-detector CT. AJNR Am J Neuroradiol.

[19] Nojiri J, Matsumoto K, Kato A (2007). The Adamkiewicz artery: demonstration by intra-arterial computed tomographic angiography. Eur J Cardiothorac Surg.

[20] Okuchi S, Okada T, Fujimoto K (2014). Visualization of lenticulostriate arteries at 3T: optimization of slice-selective off-resonance sinc pulse-prepared TOF-MRA and its comparison with flow-sensitive black-blood MRA. Acad Radiol.

[21] Okudera T, Huang YP, Fukusumi A, Nakamura Y, Hatazawa J, Uemura K (1999). Micro-angiographical studies of the medullary venous system of the cerebral hemisphere. Neuropathology.

[22] Osuafor CN, Rua C, Mackinnon AD (2022). Visualisation of lenticulostriate arteries using contrast-enhanced time-of-flight magnetic resonance angiography at 7 Tesla. Sci Rep.

[23] Saito R, Kumabe T, Inoue T (2009). Magnetic resonance imaging for preoperative identification of the lenticulostriate arteries in insular glioma surgery. Technical note. J Neurosurg.

[24] Saito R, Kumabe T, Kanamori M, Sonoda Y, Tominaga T (2010). Insulo-opercular gliomas: four different natural progression patterns and implications for surgical indications. Neurol Med Chir (Tokyo).

[25] Sanai N, Polley MY, Berger MS (2010). Insular glioma resection: assessment of patient morbidity, survival, and tumor progression. J Neurosurg.

[26] Shibahara I, Sato S, Hide T (2021). Postcentral gyrus resection of opercular gliomas is a risk factor for motor deficits caused by damaging the radiologically invisible arteries supplying the descending motor pathway. Acta Neurochir (Wien).

[27] Suzuki R, Kanamori M, Saito R, Shimoda Y, Watanabe M, Tominaga T (2023) Spontaneous shrinkage of isocitrate dehydrogenase (IDH)-mutant astrocytoma caused by intra-tumoural cyst rupture: a case report. Br J Neurosurg 1:1–5. 10.1080/02688697.2023.2170328 10.1080/02688697.2023.2170328 36722392

[28] Türe U, Yaşargil MG, Al-Mefty O, Yaşargil DC (2000). Arteries of the insula. J Neurosurg.

[29] Yoshioka K, Tanaka R, Takagi H (2018). Ultra-high-resolution CT angiography of the artery of Adamkiewicz: a feasibility study. Neuroradiology.

