# Supplementary figures and images for: Visualization of the lenticulostriate arteries, long insular arteries, and long medullary arteries on intra-arterial computed tomography angiography with ultrahigh resolution in patients with glioma
Source: Acta Neurochir (Wien). 2023 Sep 20;165(12):4213–9. doi: 10.1007/s00701-023-05794-1 (PMC10739552; doi:10.1007/s00701-023-05794-1)

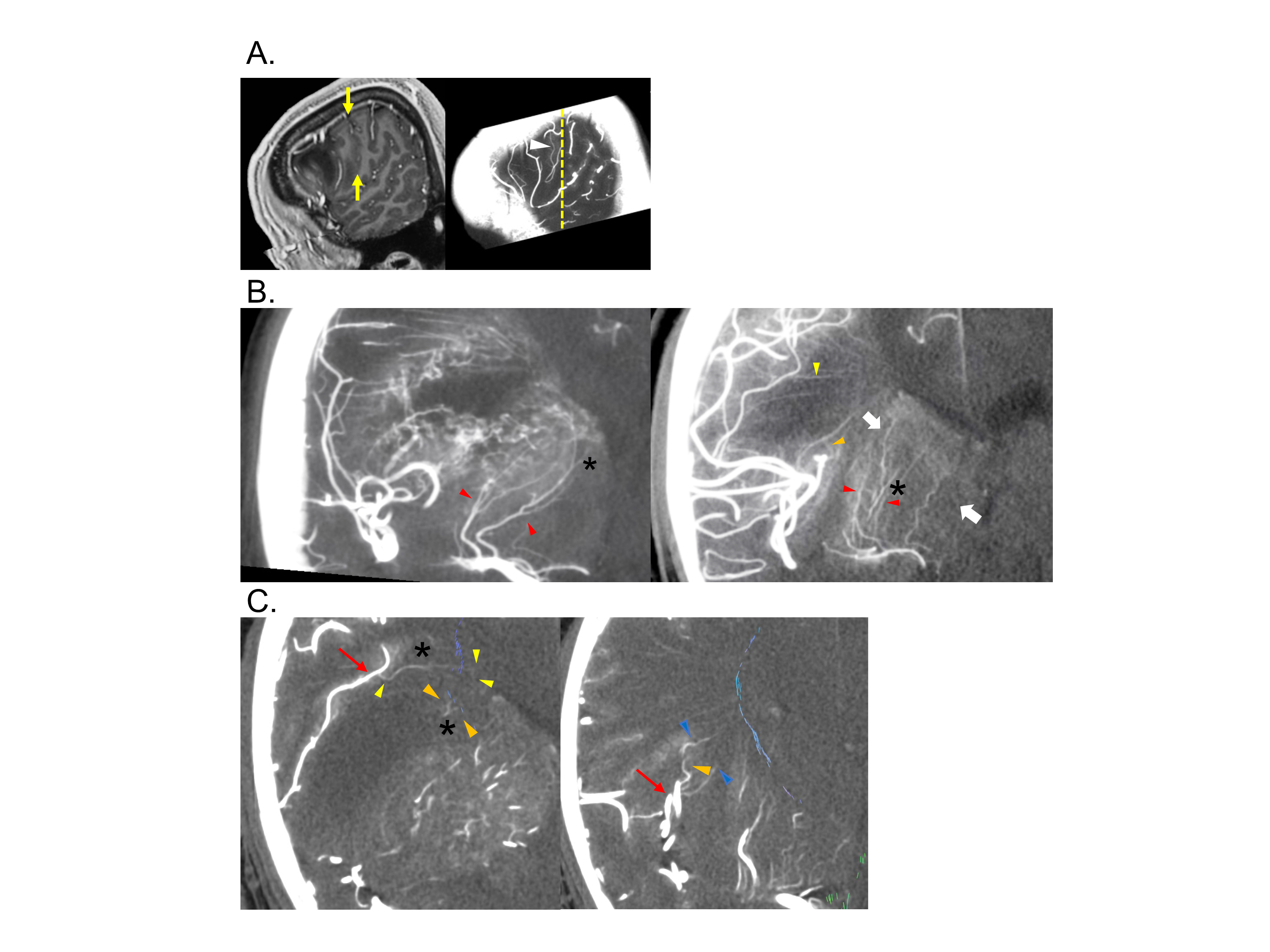

Supplement: Supplementary file 3 — Supplementary file3 Intra-arterial computed tomography (CT) angiography using ultrahigh-resolution CT (UHR-IA-CTA) in Case 1. A. Sagittal T1-weighted magnetic resonance (MR) imaging after the administration of gadolinium (Gd-T1WI) (left panel) and 20-mm-thick slab imaging of UHR-IA-CTA (right panel), thereby showing the coronal slice (yellow dashed line) for identifying the long insular artery (LIA) and long medullary artery (LMA). Based on the central sulcus (yellow arrows) on sagittal Gd-T1WI, the central artery (white arrowhead) was identified, and the coronal slice parallel to the central arteries was made on UHR-IA-CTA. B. The coronal slice with the lenticulostriate arteries (LSAs) (left panel) and with the LIA and LMA (right panel) of UHR-IA-CTA with 20-mm-thick slabs showing the distribution of the LSAs (red arrowheads), LIAs (orange arrowhead), and LMAs (yellow arrowhead). There were tortuous and dilated tumor vessels in the frontal lobe and insula, and LSAs were displaced medially by the tumor. The asterisk indicates LSA branching in the putamen and caudate nucleus. The white arrows in the right panel indicates the internal capsule. The branching, change in size, tortuosity, and density of LSA branches around the internal capsule were noted. C. The coronal slice with the LIAs and LMAs of UHR-IA-CTA with 2-mm-thick slabs, which fused to tractography of the pyramidal tract showing the association between the LIAs (orange arrowheads) and LMAs (yellow arrowheads) supplying the pyramidal tract (purple, blue and green), M2, M3, and M4 (red arrows). LIAs and LMAs directly originated from the M2–M3 junction and M4 and supplied the pyramidal tract. The vessels not connecting the middle cerebral arteries with vague enhancement were considered as the subependymal veins (blue arrowheads) that drain into the peri-insular sulcus or insular vein at the superior limiting sulcus.(TIF 2456 KB) [file 701_2023_5794_MOESM3_ESM.tif]

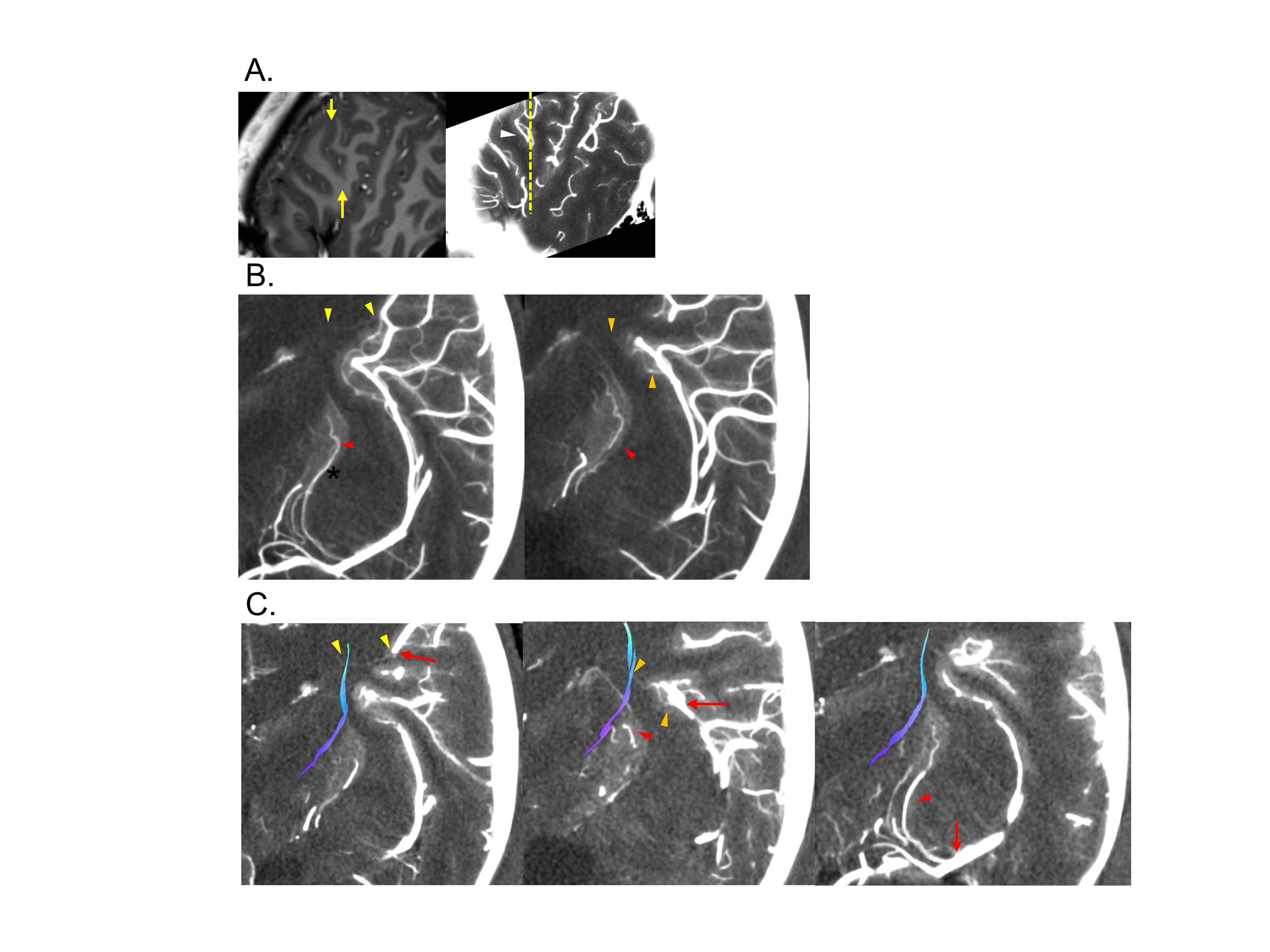

Supplement: Supplementary file 4 — Supplementary file4 Intra-arterial computed tomography (CT) angiography using ultrahigh-resolution CT (UHR-IA-CTA) in Case 2. A. Sagittal T1-weighted MR imaging after the administration of gadolinium (Gd-T1WI) (left panel) and 20-mm-thick slab imaging of UHR-IA-CTA (right panel) showing the coronal slice (dashed line) for identifying the long insular arteries (LIA) and long medullary arteries (LMA). Based on the central sulcus (yellow arrows) on sagittal Gd-T1WI, the central artery (white arrowhead) was identified, and the coronal slice parallel to the central arteries was made on UHR-IA-CTA. B. The coronal slice with the lenticulostriate arteries (LSAs) (red arrowhead), long medullary artery (LMAs) (yellow arrowheads in the left panel), and long insular artery (LIA) (orange arrowheads in the right panel) of UHR-IA-CTA with 20-mm-thick slabs showing the distribution of the LSA, LMA, and LIA. C. The coronal slice with the LMA (yellow arrowheads in the left panel), LIA (orange arrowheads in the middle panel), and proximal LSAs (red arrowhead in the middle and right panel) of UHR-IA-CTA with 2-mm-thick slab, which fused to tractography of the pyramidal tract, showing the association between the perforating arteries and pyramidal tract (purple, blue and green) and the middle cerebral arteries (red arrows). The LSAs, LIAs, and LMAs supplying the pyramidal tract originated from the M1, M2,, and M3 junction, and M4, respectively.(TIF 2390 KB) [file 701_2023_5794_MOESM4_ESM.tif]

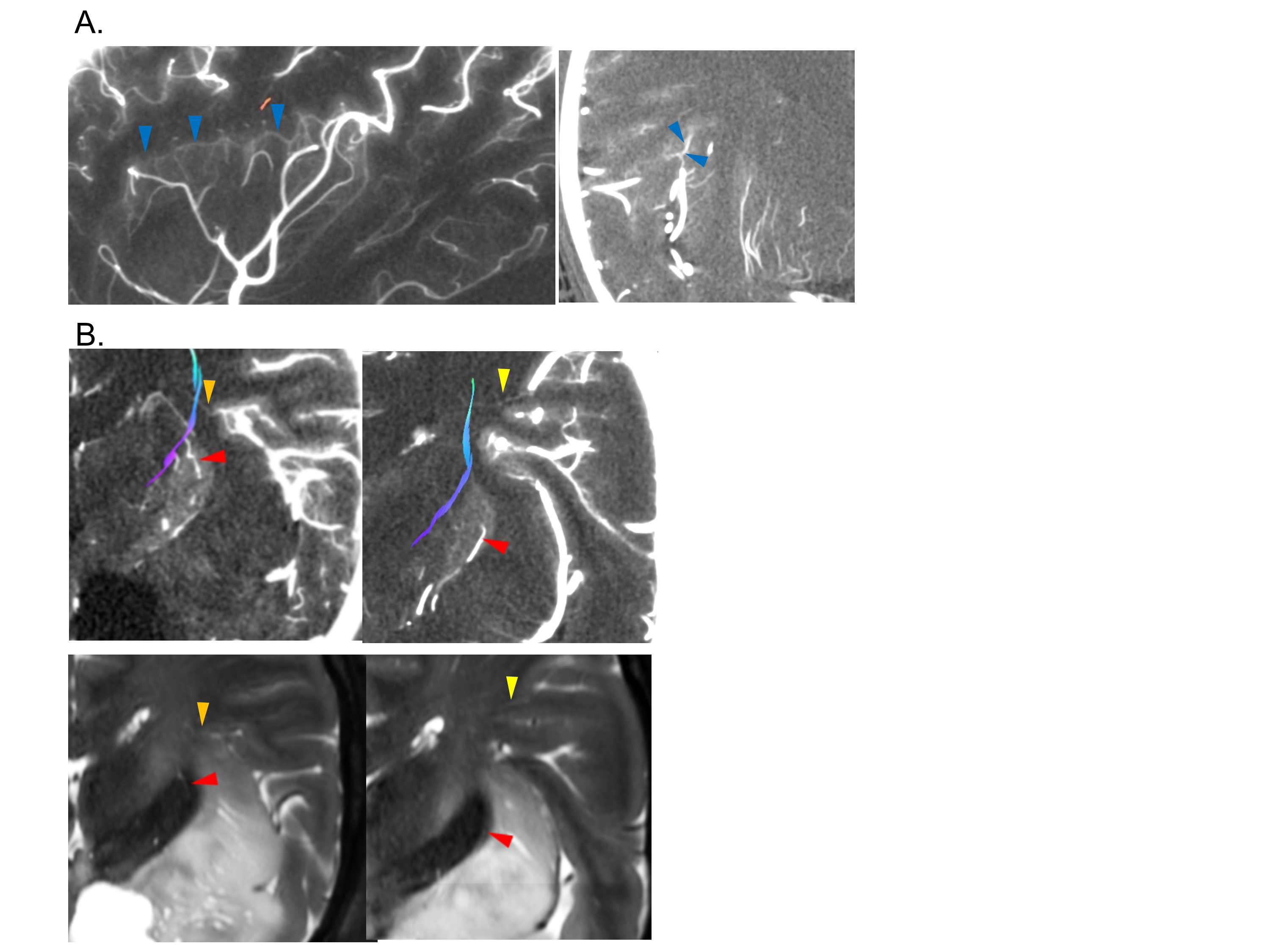

Supplement: Supplementary file 5 — Supplementary file5 The discrimination of the perforating arteries from the veins. A. Sagittal imaging (left panel) and the coronal slice parallel to the central artery (right panel) of intra-arterial computed tomography (CT) angiography using ultrahigh-resolution CT (UHR-IA-CTA) with 20-mm-thick slabs (left panel) showing the peri-insular sulcus veins along the superior limiting sulcus. These vaguely enhanced vessels were connected to the peri-insular sulcus veins along the superior limiting sulcus, but not to the M2, M3, or M4 (blue arrowheads). B. UHR-IA-CTA with 2-mm-thick slabs (upper panels) and T2-weighted imaging (T2WI) (lower panels) that is the identical cross image to UHR-IA-CTA in Case 2 showing the perivascular space around the lenticulostriate arteries (red arrowheads), long medullary arteries (yellow arrowhead), and long insular arteries (orange arrowhead) on T2WI.(TIF 2494 KB) [file 701_2023_5794_MOESM5_ESM.tif]
